# Compression of the Trachea near Its Bifurcation, by Tuberculous Bronchial Glands, with Anomalous Symptoms

**Published:** 1839-01-26

**Authors:** J. T. Sharpless


*Compression of the Trachea near its bifurcation, by tuberculous bronchial glands, with anomalous symptoms.* By J. T. Sharpless, M. D.

A child, aged six months, was attacked in April last, in apparently perfect health, with a severe convulsion in the night, which lasted for a few minutes, and went off, leaving him for six weeks with a noisy, wheezing respiration, without any cough or other indication of bad health. He then had a violent catarrh for a few days, which passed away, carrying with it all the obstruction of breathing. He remained perfectly well for some weeks, when another convulsion in the night was succeeded by the same asthmatic condition. In a few nights, another fit occurred. Upon the closest examination, no indication of disease could be discovered in the lungs, except a universal sonorous and sibilant rhonchus, occasionally mingled with a subcrepitant or mucous râle. Sound, upon percussion, clear. There was a slight cough, with some loose mucus in the trachea, but often for many hours, the respiration was perfectly dry, the *expiration* being the most difficult and noisy. He had no fever, and was generally playful through the day,—but in the night the obstruction of breathing was increased, so that he would throw himself about the bed in great distress. All the usual remedies were employed, as leeches to back and throat, sinapisms and blisters to chest, warm baths, nauseants, minute doses of calomel, etc. etc., but without any permanent relief. In about four weeks, he had four convulsions in forty-eight hours, but which, like all the others, produced no spasm of the limbs, except a mere momentary stiffening of the arms, but was confined to the chest and face, and seemed to arise from a difficulty in getting the breath. At my request, my friend Dr. Gerhard examined the case, and agreed with me in sentiment that there was no lesion in the lungs, but a general bronchial irritation, with considerable secretion of mucus, which could now be felt by the hand placed on the ribs. The cough seemed now looser, so that mucus occasionally would be brought up and swallowed; and when he cried violently, his inspirations were accompanied by a clear, loud whoop, like that of pertussis. We now gave him stimulating expectorants and antispasmodics, as assafœtida and seneka, with external friction, etc.; but there appeared little relief, however, from any remedy; and although he was very playful all day, his nights were bad, and often, for hours, entirely sleepless.* The 9th of January, 1839, he was unusually well; breathing rather better; expectoration more free, and general strength much improved; and was playing on the floor, when he became irritated by the nurse refusing to take him, and screaming, was seized with a convulsion, and died in a few seconds, at the age of fourteen months.

The child was examined eighteen hours after death, by Dr. Gerhard and myself.

On opening the thorax, and dividing the trachea at the distance of a little more than half an inch above its bifurcation, we observed a tumour which completely enclosed the trachea, and extended around the point of bifurcation. Its whole length was two inches, and it was about an inch in breadth. At first sight, the tumour seemed to be homogeneous; but, upon examination, it was evidently composed of a cluster of lymphatic glands, closely united together, but contained in distinct capsules. They were of a pale whitish colour, and contained grains of tuberculous matter. The largest of them offered a commencement of softening at its centre. The aorta was slightly compressed by the mass, as well as the œsophagus. The trachea was completely changed in form, and compressed into an angular appearance; its calibre not more than one-half the natural size.

The interior of the larynx was in the normal state; but the trachea was filled with thick whitish mucus, and its membrane was reddened and thickened. The same mucus and appearance of the membrane continued throughout the bronchial tubes. The tissue of the lungs was healthy, with the exception of a few lobules, which were reddish and impermeable to the air. There were also some scattered tuberculous granulations, evidently of recent origin.

This case is curious, and illustrates many obscure points. Tuberculous alterations of the bronchial glands are, perhaps, the most common of all lesions observed in the bodies of children; but it is so rare to meet with glands enlarged to the extent observed in the present case, that in many hundred examinations at the Children's Hospital of Paris, Dr. Gerhard did not meet with a single case in which the compression of the trachea was equally great. The bronchitis was strictly secondary, and of itself was unimportant. The dyspnœa was not constant, but paroxysmal,—a peculiarity which may be observed in most cases of tumours pressing upon the trachea or bronchi. Convulsions are a common symptom attendant upon the attacks of dyspnœa.

The existence of enlarged bronchial glands cannot be known with certainty, unless they happen to extend anteriorly in such a manner as to force the lungs aside; we then recognise their presence by the dulness of percussion. In the present case the tumour was covered by the lung, and the dulness was not greater than usual.

If the child had not been carried off by an accidental convulsion, recovery might have followed; that is, the bronchial glands were already adherent to the trachea, and would probably have softened and discharged into the trachea by an ulcerated opening. There would still have remained much danger from the tuberculous disease which had already commenced in the lungs.

---

### REMARKS ON DELIRIUM TREMENS.

By William H. Klapp, M. D.

To the Editors of the Medical Examiner.

*Gentlemen:*—In the last number but one of your very valuable periodical, you have reported a lecture of Dr. Coates on delirium tremens; and, in casting my eye over it, I perceive that the Doctor has been betrayed into an error re-

* There was occasionally a difficulty in swallowing, requiring a second effort to pass his food into the stomach, which his mother thought arose from a sore throat, and mentioned it.

